# Supplementary material for: Successional Shifts in Soil C:N:P stoichiometry and its environmental controls during forest restoration in Huize County, Qujing, Yunnan Province
Source: PLoS One. 2026 Aug 21;21(8):e0356261. doi: 10.1371/journal.pone.0356261 (PMC13496410; doi:10.1371/journal.pone.0356261)
Supplement: Supplementary file 2 [file pone.0356261.s004.zip › Data and Code/Mixed Models/ANOVA_Plots/Interaction_C_N.pdf]

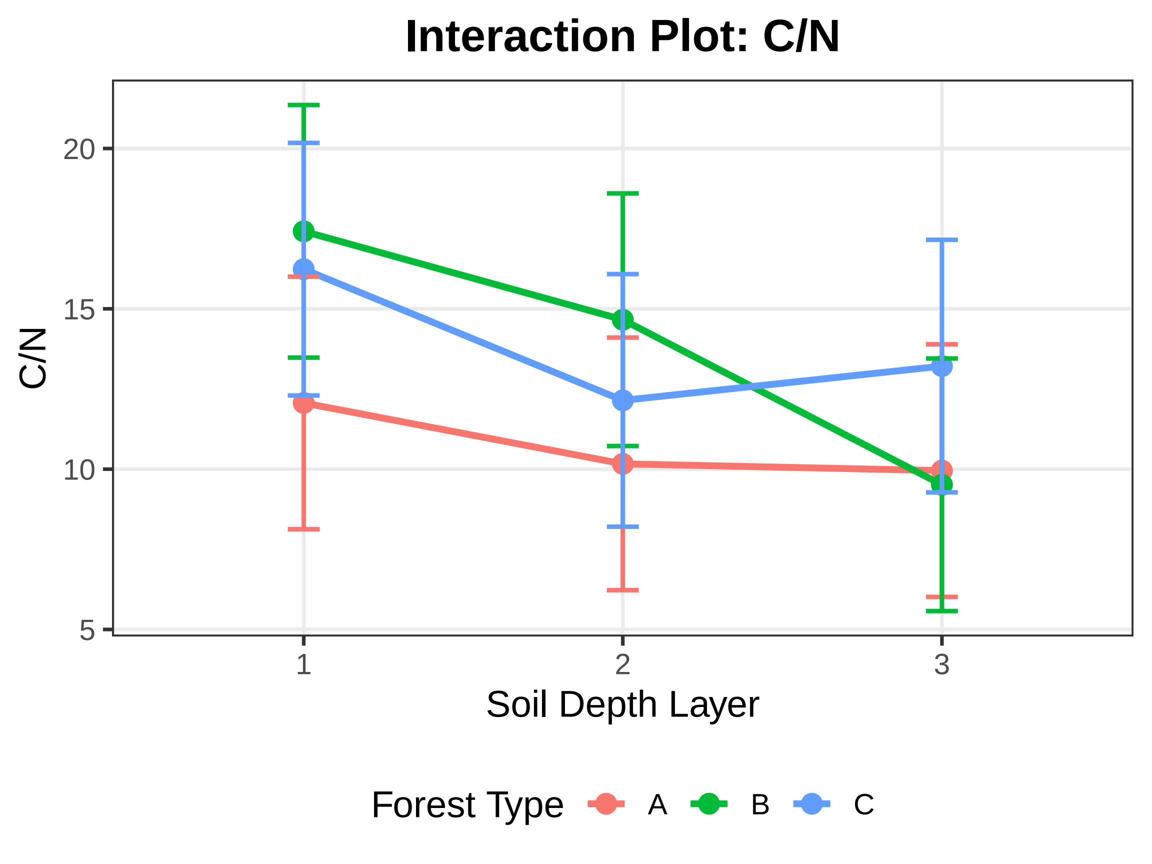

Interaction Plot: C/N
C/N
20
15
10
5
1
2
3
Soil Depth Layer
Forest Type
A
B
C
